# Familial multifocal micronodular pneumocyte hyperplasia with a novel splicing mutation in *TSC1*: Three cases in one family

**DOI:** 10.1371/journal.pone.0212370

**Published:** 2019-02-22

**Authors:** Tetsuaki Shoji, Satoshi Konno, Yo Niida, Takahiro Ogi, Masaru Suzuki, Kaoruko Shimizu, Yasuhiro Hida, Kichizo Kaga, Kuniaki Seyama, Tomoaki Naka, Yoshihiro Matsuno, Masaharu Nishimura

**Affiliations:** 1 Department of Respiratory Medicine, Faculty of Medicine and Graduate School of Medicine, Hokkaido University, Sapporo, Hokkaido, Japan; 2 Center for Clinical Genomics, Kanazawa Medical University Hospital, Ishikawa, Japan; 3 Department of Cardiovascular and Thoracic Surgery, Faculty of Medicine and Graduate School of Medicine, Hokkaido University, Sapporo, Hokkaido, Japan; 4 Division of Respiratory Medicine, Juntendo University Faculty of Medicine and Graduate School of Medicine, Tokyo, Japan; 5 Division of Pathology, National Cancer Center Hospital, Tokyo, Japan; 6 Department of Surgical Pathology, Hokkaido University Hospital, Sapporo, Hokkaido, Japan; Universita degli Studi di Verona, ITALY

## Abstract

Multifocal micronodular pneumocyte hyperplasia (MMPH) is a rare pulmonary disease, generally manifesting as a tuberous sclerosis complex (TSC), characterised by multiple, small ground-glass nodular shadows on chest computed tomography (CT). Histological examination typically reveals multicentric, well-demarcated, nodular type II pneumocystic growth. Herein, we describe three cases of this rare pulmonary disease occurring within one family. Using reverse transcription polymerase chain reaction (RT-PCR) and direct DNA sequencing, we identified a novel germline mutation, a point mutation in *TSC1* intron 5, which yielded a splice variant and loss of function of *TSC1*. Furthermore, immunohistochemical staining indicated the expression of phospho-p70S6K and phospho-4E-BP1, suggesting that *TSC1* function was impaired by the novel gene mutation in MMPH cells.

## Introduction

Multifocal micronodular pneumocyte hyperplasia (MMPH) is a rare pulmonary manifestation of tuberous sclerosis complex (TSC), characterised by multiple solid nodules or nodular ground glass opacities ranging from 2 to 10 mm, randomly distributed throughout the lungs, as revealed via high-resolution computed tomography (HRCT) [1]. Histologically, MMPH is characterised by multicentric, well-demarcated nodular growth of type II pneumocytes [2]. Thus, both radiologically and histologically, atypical adenomatous hyperplasia (AAH) and well-differentiated adenocarcinoma should always be considered a differential diagnosis of MMPH [3, 4]. Some reports have described cases of MMPH wherein clinical features associated with TSC were not observed [2, 5].

TSC, an autosomal dominant disease caused by a mutation in either *TSC1* or *TSC2* [6, 7], is characterised by the development of hamartomas in various organs, such as the brain, lungs, and kidneys [8]. The diagnostic criteria for TSC were established in 1998, 2004, and 2012 [8–10]. A recent molecular analysis revealed that a loss-of-function mutation in either *TSC* gene, both tumour-suppressor genes, causes benign neoplastic proliferation [11, 12]. However, the criteria in 1998 and 2004 included only clinical symptoms owing to the lack of advanced technology for genetic analysis, and it was difficult to detect *TSC1/2* mutations. Technological advancements made it possible to detect mutations in *TSC1/2* in 75–90% of patients [13], and genetic analysis was included in the diagnostic criteria in 2012 [8]. Interestingly, TSC patients who share the same mutations in *TSC1/2* often present different clinical manifestations and clinical outcomes [14, 15]. Therefore, some TSC-related lesions appear to require an additional event in each organ in addition to a mutation in one *TSC1/2* allele. An example of one such event is loss of heterozygosity (LOH), which inactivates the remaining wild-type allele (the second hit) [16]. LOH contributes to the pathogenesis of renal angiomyolipoma, although monoallelic inactivation of *TSC1/2* seems to be sufficient for cortical tubers [17–20]. A previous study reported that LOH occurs frequently in MMPH [3], whereas in several other reports, LOH did not occur in this condition [21, 22].

Herein, we describe three cases of this rare pulmonary disease within a single family. Although MMPH is mostly associated with TSC, and *TSC1/2* mutations are probably involved in MMPH pathogenesis, familial aggregation of MMPH has not been reported. Through reverse transcription polymerase chain reaction (RT-PCR) and direct DNA sequencing, we identified a novel germline mutation, a point mutation in *TSC1* intron 5, which yielded a splice variant accompanied by loss of function of *TSC1*. We evaluated LOH in *TSC1* in lung tissues to investigate the role of LOH in MMPH development, which was followed by immunohistochemical analysis.

## Materials and methods

### Patients

Three MMPH patients in a single family and one healthy relative were assessed at Hokkaido University Hospital; the three patients comprised the daughter (patient 1), mother (patient 2), and son (patient 3), and the healthy individual was the father. Patients 1 and 3 fulfilled the diagnostic criteria for TSC per the latest clinical diagnostic criteria of 2012 [8].

### Histological and immunohistochemical examination of lung specimens

Lung specimens were fixed in 10% formaldehyde, embedded in paraffin, and stained with haematoxylin-eosin (HE) and Elastica-Masson (EMa). Specimens from patients 1 and 2 were examined immunohistochemically using the following commercially available antibodies: anti-p53 (DO-7, 1/200 dilution; Leica Biosystems, Nussloch, Eisfeld, Germany), anti-CEA (II-7, 1/50 dilution; Dako, Santa Clara, CA, USA), anti-Ki-67 (MIB1, 1/200 dilution; Dako), anti-melanosome (HMB45, 1/100 dilution; Dako), anti-phospho-AKT (#3787, 1/25 dilution; Cell Signaling Technology, Danvers, MA, USA), anti-phospho-p70S6K (#9206, 1/50 dilution; Cell Signaling Technology), and anti-phospho-4E-BP1 (#2855, 1/25 dilution; Cell Signaling Technology) antibodies.

### Gene analysis

DNA and RNA were extracted from peripheral blood leukocytes, and cDNA was synthesised in accordance with standard methods. DNA was extracted from formalin-fixed, paraffin-embedded tissues using a WaxFree DNA Extraction Kit (Trimgen, Sparks Glencoe, MD, USA). Sequential *TSC1/2* mutational screening was performed. To detect point mutations, CEL nuclease-mediated heteroduplex incision with polyacrylamide gel electrophoresis and silver staining (CHIPS) analysis were performed for all coding exons of both *TSC1* and *TSC2* genes [23]. If no mutations were detected upon CHIPS screening, we performed long-range PCR to detect large gene deletions or duplications in *TSC1* or *TSC2* [24]. In addition, we performed next-generation sequencing clinically complemented at Hokkaido University Hospital using genomic DNA from peripheral blood; we performed targeted amplicon exome sequencing for 160 cancer-related genes including *TSC1/2* using the Illumina MiSeq sequencing platform (Illumina, San Diego, CA, USA), and the sequencing data were analysed using an original bioinformatics pipeline called GenomeJack (Mitsubishi Space Software, Tokyo, Japan).

If no mutations were detected by these approaches, we performed RT-PCR to detect splice variants resulting from deep intronic mutations. Several PCR primer sets were designed to encompass the entire coding region for both genes. Following the identification of *TSC1* splice variants, insertion of a part of the intron 5 sequence was detected in abnormal splice products. We determined the sequence of the entire *TSC1* intron 5 in genomic DNA.

LOH analysis of lung lesions was performed via PCR-restriction fragment length polymorphism (RFLP) analysis. PCR-RFLP was performed using *SspI*, which selectively cleaved the wild type allele. After agarose gel electrophoresis and ethidium bromide staining, the density of each band was calculated using ImageJ software (NIH, Bethesda, MD, USA).

Primer sequences and PCR conditions used for all experiments are available on request. For *TSC1* sequencing, DNA and cDNA were evaluated in accordance with GenBank accession numbers NG_012386.1 and NM_000368.4, respectively.

### Ethics and consent

This study was approved by the ethics committees of the Research Review Board of Hokkaido University (approval no. 14–057), Hokkaido University Hospital (approval no. 017–0177), and Kanazawa Medical University Hospital (approval no. 111). Written informed consent was obtained from all three patients and three healthy controls directly by the physicians (K.I., T.S., and S.K.) in accordance with the tenets of the Declaration of Helsinki. All methods were performed in accordance with the relevant guidelines and regulations of Hokkaido University, Hokkaido University Hospital, and Kanazawa Medical University Hospital.

## Results

### Case presentation

**Fig 1:**
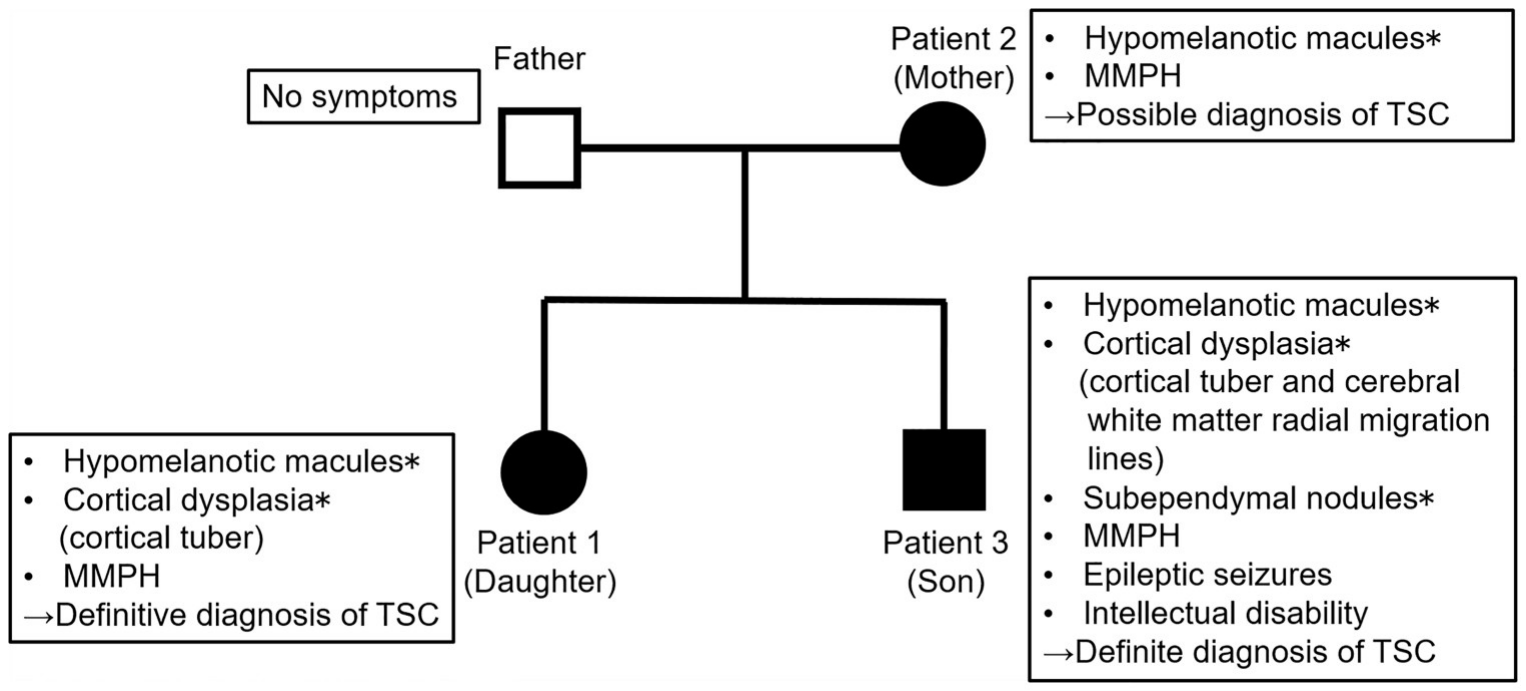
Relationships among and characteristics of patients in this study. The three patients had multifocal micronodular pneumocyte hyperplasia. Patients 1 and 3 (daughter and son) were diagnosed with tuberous sclerosis complex in accordance with the 2012 diagnostic criteria. The father of patients 1 and 3 did not display any clinical indications of a tuberous sclerosis complex. *Major features included in the 2012 diagnostic criteria.

Three MMPH patients in a single family and one healthy relative were assessed; the three patients comprised the daughter (patient 1), mother (patient 2), and son (patient 3), and the healthy individual was the father. Patients 1 and 3 fulfilled the diagnostic criteria for TSC per the latest clinical diagnostic criteria of 2012 [8]. Demographic information of the family is summarised in Fig 1.

**Fig 2:**
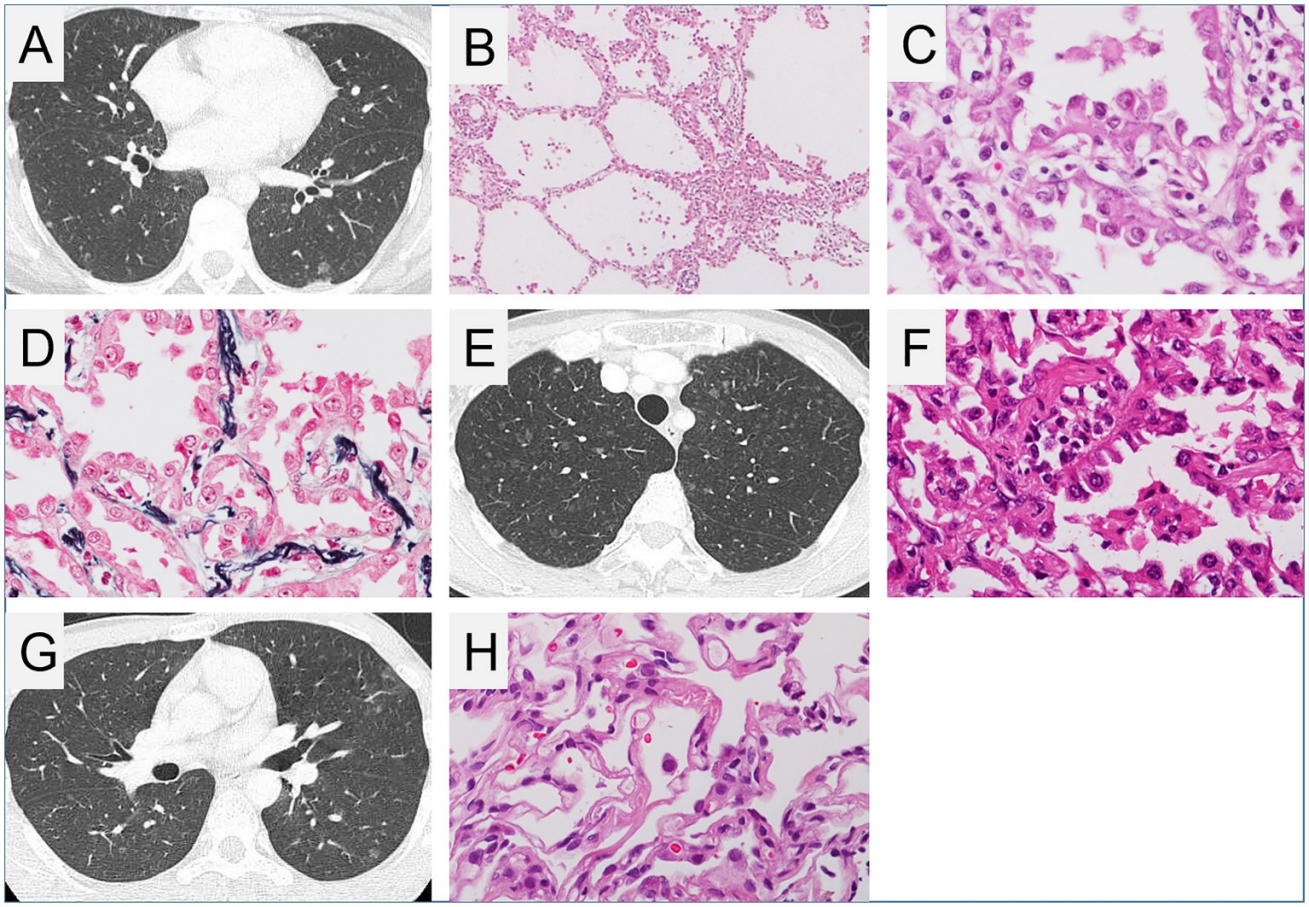
Radiological and histopathological findings of multifocal micronodular pneumocyte hyperplasia. (A) Chest high-resolution computed tomography (HRCT) image of patient 1 (daughter) showed several ground glass nodules measuring 1–7 mm in size. Cystic changes suggestive of lymphangioleiomyomatosis (LAM) were not observed. (B–D) Histopathological images of multifocal micronodular pneumocyte hyperplasia (MMPH) of patient 1. (B) Low-powered view (×20), haematoxylin and eosin (HE) staining. There were well-demarcated, nodular lesions ranging from 2 to 5 mm in the pulmonary parenchyma. (C) High-powered view (×200), HE staining. Alveolar structure was maintained. Enlarged type II pneumocytes lining the thickened alveolar septa had abundant eosinophilic cytoplasm and spherical nuclei with mild nuclear atypia. (D) High-powered view (×200), Elastica-Masson staining. The alveolar septa were thickened by proliferation of elastic fibres. (E) Chest HRCT image of patient 2 (mother) showed several micro ground glass nodules. (F) Histopathological image of lung specimen of patient 2. High-powered view (×200), HE staining. Multifocal well-demarcated nodular lesions were observed. Enlarged cuboidal type II pneumocytes had swollen nuclei. The alveolar septa were thickened by fibrous proliferation. (G) Chest HRCT image and (H) histopathological image, high-powered view (×200), HE staining, of the lung lesion of patient 3 (son) showed findings consistent with MMPH.

Patient 1 was a 30-year-old woman referred to our hospital because of multiple ground-glass opacities (GGOs) that were revealed upon chest computed tomography (CT), which was performed to investigate the cause of her prolonged mild cough. HRCT revealed several nodular shadows measuring 1–7 mm in the bilateral upper and middle lung fields (Fig 2A). These shadows were initially considered to represent AAH or well-differentiated adenocarcinoma. Video-assisted thoracic surgery (VATS) lung biopsy was performed on the left upper lobe (S6) of the lung. Diffuse multiple nodular GGOs, identified via CT, comprised uniform proliferation of type II pneumocytes with bland nuclear morphology along the alveolar septa, as revealed through histological analysis (Fig 2B–2E). The alveolar septa also showed fibrous thickenings, increased numbers of elastic fibres, and aggregation of alveolar macrophages. Immunohistochemistry revealed that all proliferating alveolar epithelial cells did not express HMB45, carcinoembryonic antigen (CEA), and Ki-67, while some cells expressed p53. Therefore, we diagnosed MMPH upon histopathological evaluation. Although the patient had small (roughly 2 cm × 2 cm) hypomelanotic macules on her left thigh, left upper arm, and epigastrium, other clinical findings suggestive of TSC were not reported. We previously reported this woman as an MMPH patient without diagnosis of TSC [25] (ref. in Japanese).

Four years after having diagnosed MMPH in patient 1, her 55-year-old mother (patient 2) was referred to our hospital owing to an abnormal chest X-ray following physical examination. Chest HRCT revealed bilateral diffuse nodular GGOs extending from the upper to lower lobes (Fig 2E). VATS lung biopsy was performed on the right upper and lower lobes. Microscopic examination revealed proliferation of type II pneumocytes along with fibrous thickenings of the alveolar septa, as observed in patient 1, thereby suggesting MMPH (Fig 2F). Patient 2 had very small (5 mm × 5 mm) hypomelanotic macules on her upper limb and back; however, no other clinical findings suggestive of TSC were observed.

**Fig 3:**
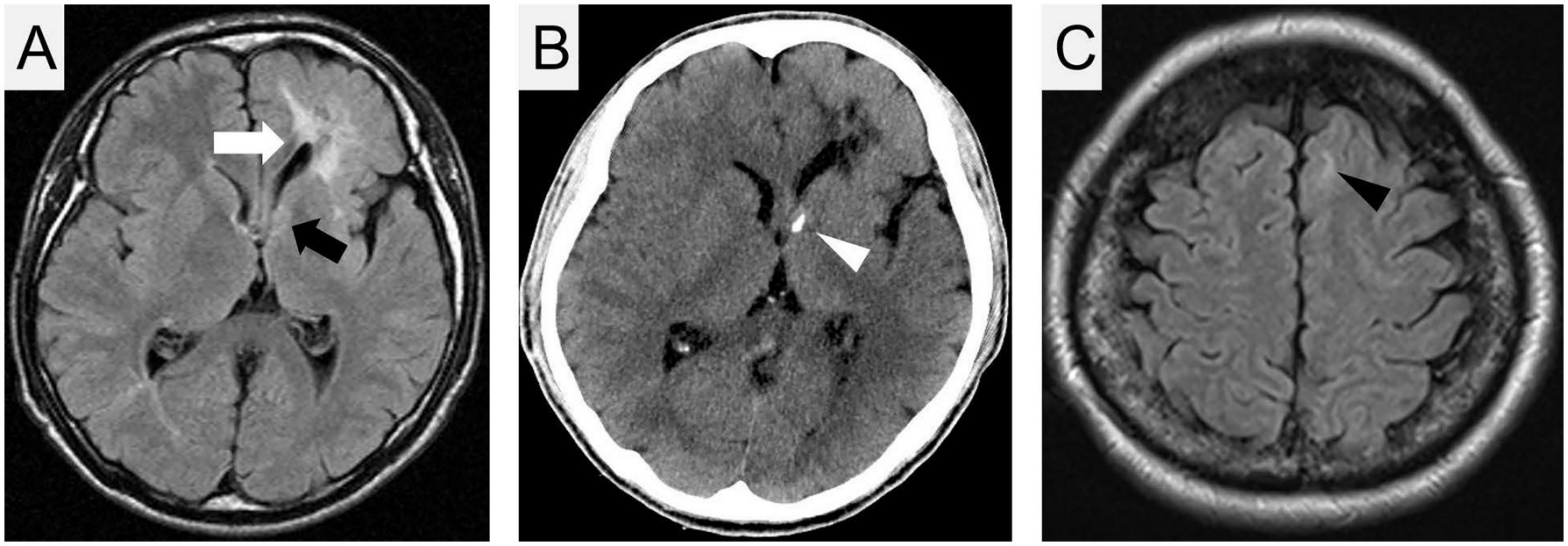
Brain imaging. Axial magnetic resonance imaging (MRI; T2 fluid-attenuated inversion recovery) (A) and computed tomography (CT) imaging (B) of patient 3. The high-intensity area near the left lateral ventricle represents cortical dysplasia (tubers and radial migration lines; white arrow). The high-intensity area, which was observed in MRI (black arrow) with calcification in CT (white arrowhead) near the left Monro foramen, represents a subependymal nodule. (C) MRI (T2 fluid-attenuated inversion recovery) of patient 1. The high-intensity area in the left frontal lobe represents a cortical tuber (black arrowhead).

Owing to the diagnosis of MMPH in patients 1 and 2, we recommended chest screening for the patient’s 33-year-old brother (patient 3) at our hospital. He had presented with epileptic seizures at 10 years of age and had been prescribed antiepileptics at a local clinic. The results of the third-edition Wechsler Adult Intelligence Scale (WAIS-III) indicated that the patient’s complete IQ score was 58, indicating mild mental retardation. Although patient 3 did not experience any respiratory symptoms, HRCT revealed diffuse bilateral nodular GGOs extending from the upper to lower lobes (Fig 2G). Transbronchial lung biopsy specimens from the right upper lobe revealed multifocal, well-demarcated, nodular lesions indicating the proliferation of type II pneumocytes with mild fibrous thickenings of the alveolar septa, consistent with MMPH (Fig 2H). He had several hypomelanotic macules on his right thigh (largest size: 25 cm × 4 cm). T2-weighted magnetic resonance imaging (T2-MRI) revealed high-intensity areas in the right frontal and occipital lobes and in the left frontoparietal lobe, which were thought to represent cortical dysplasia (tubers and radial migration lines; Fig 3A). Brain CT revealed coarse nodular calcification near the foramen of Monro in the anterior region of the right ventricle, thereby suggesting the presence of subependymal nodules (Fig 3B). Thus, a definitive diagnosis of TSC was made [8].

Following the diagnosis of TSC in patient 3, the clinical signs of TSC were systematically re-evaluated in patients 1 and 2. In patient 1, careful re-evaluation of the brain T2-MRI revealed a small high-intensity area in the left frontal lobe, which was thought to represent cortical tubers (Fig 3C). Together with the presence of small hypomelanotic macules and MMPH, patient 1 was diagnosed with definite TSC per the current criteria published in 2012 [8]. Patient 2 did not display any clinical signs suggestive of TSC, except for MMPH and small hypomelanotic macules. Therefore, patient 2 did not fulfil the current TSC criteria [8].

Chest HRCT of the father of patients 1 and 3 (the husband of patient 2), i.e. the healthy control individual, did not reveal any abnormalities. No clinical TSC-related signs were observed upon direct interview or systemic evaluation.

### Analysis of germline mutations in *TSC1* and *TSC2*

We analysed germline mutations in *TSC1* and *TSC2* using DNA and RNA extracted from peripheral blood leukocytes. Through CHIPS and direct sequencing [23], we detected a nonsynonymous amino acid substitution in *TSC1*, p.Gln654Glu, in patients 1 and 3 (S1 Dataset). This substitution reportedly has a conflicting interpretation regarding its pathogenicity in ClinVar (https://www.ncbi.nlm.nih.gov/clinvar/); the healthy father had the same substitution. However, patient 2 (the mother) did not harbour this mutation. Therefore, we concluded that p.Gln654Glu might not have pathological significance. Previously, long PCR has been reported to be useful for finding large deletions and duplications [24]. However, long PCR for *TSC1* and *TSC2* in this study revealed that there were no large deletions or duplications (S1 Dataset). Furthermore, upon next-generation sequencing, no pathogenic genomic alteration was detected in all exons of 160 cancer-related genes (S2 Dataset).

**Fig 4:**
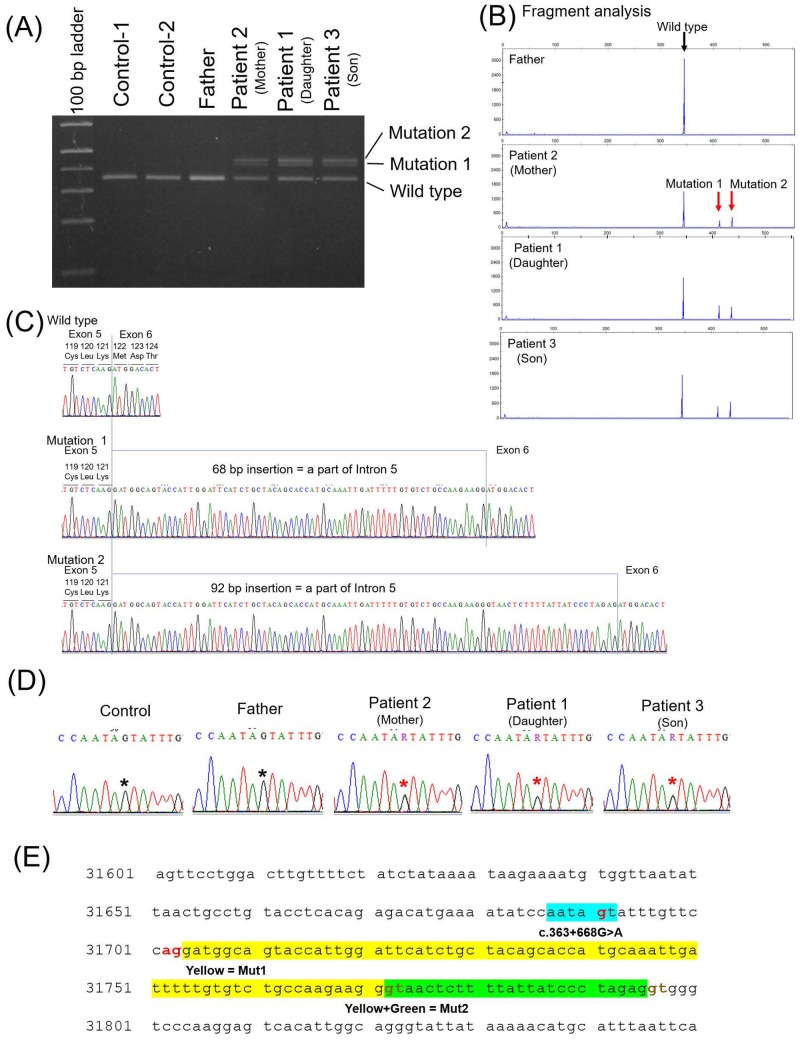
Analysis of RNA and introns of *TSC1* and *TSC2*. Three patients (patients 1, 2, and 3), their healthy father (husband of patient 2), and two healthy controls were assessed. Total RNA was extracted from peripheral blood lymphocytes and template DNA was synthesised. (A, B) Reverse transcription-PCR (RT-PCR) with the template DNA and primer pair covering exons 5–7 of *TSC1*. Full-length agarose gel electrophoresis of RT-PCR products is shown in S1 Fig. In lanes for all three patients, two additional, longer amplification product bands were observed (mutations 1 and 2). (C) Direct DNA sequencing with products amplified using RT-PCR revealed an insertion of 68 base pairs (bp) (mutation 1) or 92 bp (mutation 2) of a part of the intron 5 sequence between exons 5 and 6. (D) The sequences of a part of intron 5 for the three patients and their father were detected via direct sequencing of DNA extracted from peripheral blood. The three patients harboured point mutations (c.363+668G>A) in the heterojunction. (E) A section of intron 5. Intron 5 consists of 2096 bp in total. A segment of the sequence of intron 5 of *TSC1* including the point mutation was detected by direct DNA sequencing. Sixty-eight base pairs (yellow) and 92 bp (yellow and green) were inserted between exons 5 and 6, as shown in (C). The 6-bp sequence including the point mutation (light blue) may serve as a new branch point. AG (red) immediately upstream of the inserted sequence may function as the splice acceptor site, and 2 GT (brown) after the inserted sequence may function as the splice donor sites. Therefore, the point mutation may cause splicing anomalies. Because 68 and 92 are not multiples of 3, the splicing anomaly may cause a frame shift and loss of function of *TSC1* in the three patients.

Subsequently, we analysed splicing anomalies in the three patients and three healthy controls (the father and two additional healthy subjects). RT-PCR for exons 5–7 of *TSC1* revealed two additional long amplification products in all three patients (mutations 1 and 2 in Fig 4A and 4B). This suggested that two types of abnormal splicing occurred in this region. Hence, we cloned RT-PCR products and determined their sequences. Consequently, 68 or 92 base pairs (bp) of a part of intron 5 were inserted between exons 5 and 6 (Fig 4C). To identify the genetic abnormality resulting in this splicing anomaly, we sequenced the entire intron 5. This revealed that all three patients had a heterozygous single-base substitution (c.363+668G>A) in intron 5 (Fig 4D). Intron 5 is 2096 bp, and Fig 4E shows the portion of intron 5 that included this point mutation. The sequence of six bases AATAAT containing the point mutation functions as a new branch point resulting in the splicing anomaly [26]. An AG sequence immediately upstream of the newly inserted sequence is also a potential splice acceptor site. Additionally, two sites, specifically a GT sequence after 68 and 92 bp, can serve as splice donor sites. These two abnormal mRNAs caused frame shifts and functional loss of *TSC1* in the germline of all three patients. Conversely, this mutation was not identified in the three healthy controls.

### LOH analysis of lung tissue

**Fig 5:**
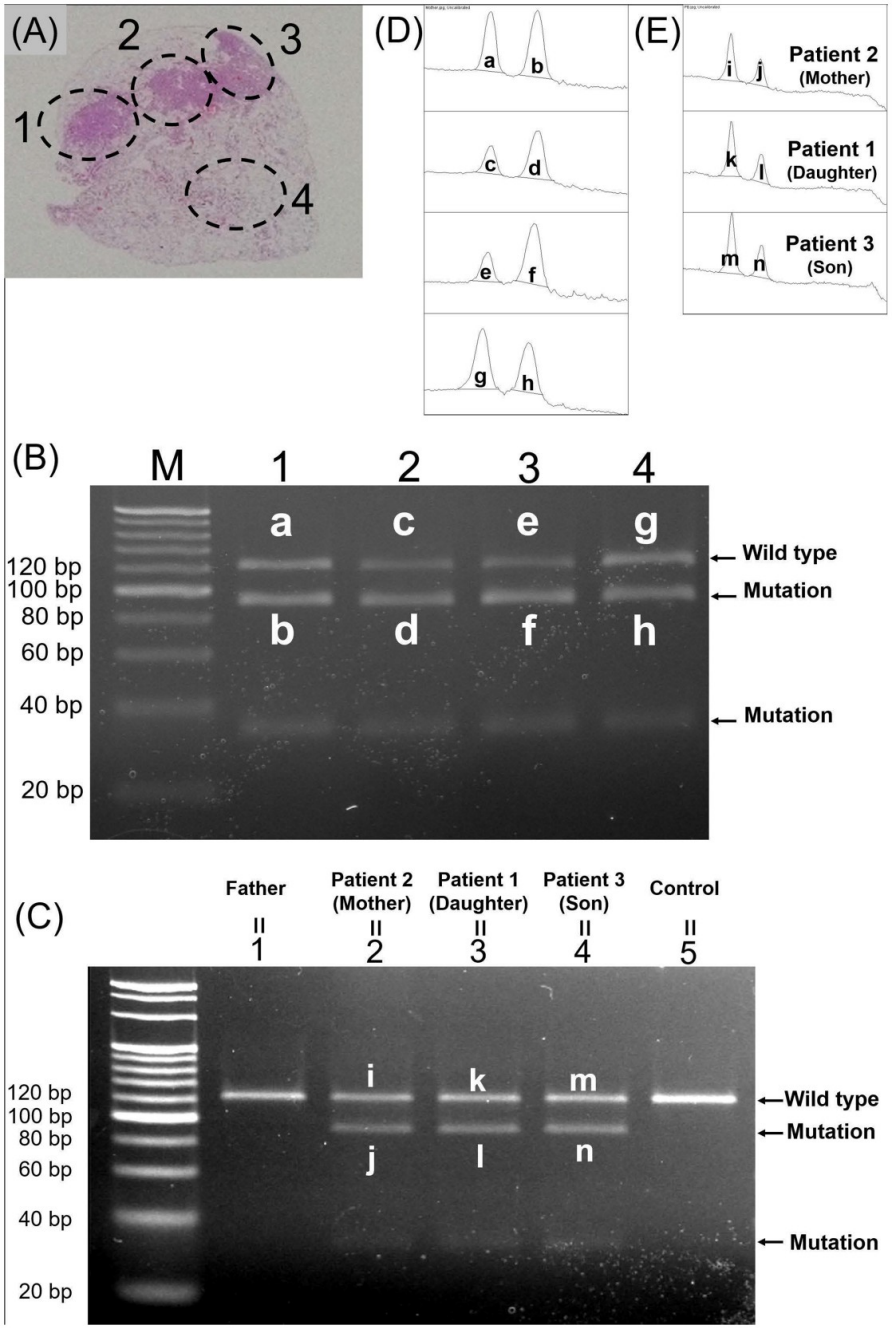
Loss of heterozygosity (LOH) analysis of lung tissue from patient 2 (mother). Polymerase chain reaction-based restriction fragment length polymorphism (PCR-RFLP) revealed that mutant alleles increased due to LOH in all lung lesions of patient 2 (mother). (A) DNA was extracted from four lesions in paraffin-embedded lung tissue obtained via video-assisted thoracic surgery lung biopsy. Lesions 1–3 included numerous multifocal micronodular pneumocyte hyperplasia (MMPH) cells, and lesion 4 included scant MMPH cells. (B) PCR-RFLP analysis of lung lesions. Full-length agarose gel electrophoresis of RT-PCR products is shown in S2 Fig. Lane numbers match the lesion numbers in (A). In all four lanes, there were bands indicating 30- and 91-bp fragments cleaved by *SspI*. (C) PCR-RFLP analysis of peripheral blood DNA from the three patients, the father, and one healthy control. Full-length agarose gel electrophoresis of RT-PCR products is shown in S3 Fig. Lanes 1–5 indicate the father, patient 2, patient 1, patient 3, and the healthy control, respectively. In the three lanes for the patients, there were 30- and 91-bp bands indicating the mutant alleles. (D, E) Densitometry analysis of bands in lung lesion DNA (D) and peripheral blood DNA (E). Letters indicating the area match those in (B, C). The area of each band determined via densitometric analysis is shown in Tables 1 and 2.

We investigated the occurrence of LOH in lung tissues of patients 1 and 2, from whom sufficient lung tissue specimens were obtained via VATS lung biopsy. Four lesions of lung tissues were analysed for each patient. Fig 5 and Table 1 illustrate the LOH analysis for patient 2 (mother). DNA was extracted from paraffin-embedded lung tissues from four lesions of each patient; three had high cell density and appeared to include many tumour cells (lesions 1–3 in Fig 5A), and one had low cell density (lesion 4 in Fig 5A). DNA was amplified with primer pairs covering a part of intron 5, including the point mutation site.

**Table 1:** Densitometric analysis of lung lesions in patient 2 (mother).

Band	Lesion no.	Wild type or mutation	Area	Area-Mut/Area-WT of each lesion
**a**	1	Wild type	7074.761	
**b**	1	Mutation	9345.075	1.321
**c**	2	Wild type	3243.861	
**d**	2	Mutation	7043.832	2.171
**e**	3	Wild type	3457.276	
**f**	3	Mutation	9123.489	2.638
**g**	4	Wild type	9042.731	
**h**	4	Mutation	7262.368	0.803

Letters indicating bands match those in Fig 5B and 5D. Numbers indicating lesions match those in Fig 5A and 5B.

We performed PCR-RFLP analysis and densitometric analysis to compare DNA from lung lesions and from peripheral blood. Peripheral blood DNA was extracted from the three patients, the father, and one healthy control individual. For PCR-RFLP, we used the restriction enzyme *SspI*, which cleaves the mutant sequence (AATATT), including the point mutation, and does not cleave the wild-type sequence AGTATT. The amplification product was 121 bp. *SspI* cleaved the mutant amplification product into 90 and 31 bp fragments. The results of PCR-RFLP of DNA from the lung lesions of patient 2 are shown in Fig 5B. We also analysed peripheral blood DNA from the three patients, the father, and one healthy control individual, using PCR-RFLP with *SspI* (Fig 5C). We then measured the area of the bands of the wild-type product and the larger mutant product from lung lesions and peripheral blood via densitometric analysis using ImageJ software (Fig 5D and 5E, Tables 1 and 2). In each lesion, the mutant area/wild-type area was greater than 0.58 (Table 1). In peripheral blood DNA, the mutant area/wild-type area for each patient was approximately 0.58 (Table 2). These data indicated that the proportion of mutant alleles increased owing to LOH generation in each lesion. For patient 1, PCR-RFLP analysis revealed that LOH was generated similarly in all lung lesions examined (S4 Fig and S1 Table).

**Table 2:** Densitometric analysis of DNA from peripheral blood of each patient.

Band	Patient	Wild type or mutation	Area	Area-Mut/Area-WT of each patient
**i**	Patient 2 (mother)	Wild type	3829.619	
**j**	Patient 2 (mother)	Mutation	2258.497	0.589
**k**	Patient 1 (daughter)	Wild type	4323.083	
**l**	Patient 1 (daughter)	Mutation	2508.205	0.580
**m**	Patient 3 (son)	Wild type	4836.690	
**n**	Patient 3 (son)	Mutation	2810.497	0.581

Letters indicating bands match those in Fig 5E.

### Functional analysis of *TSC1* mutation via immunohistochemistry

**Fig 6:**
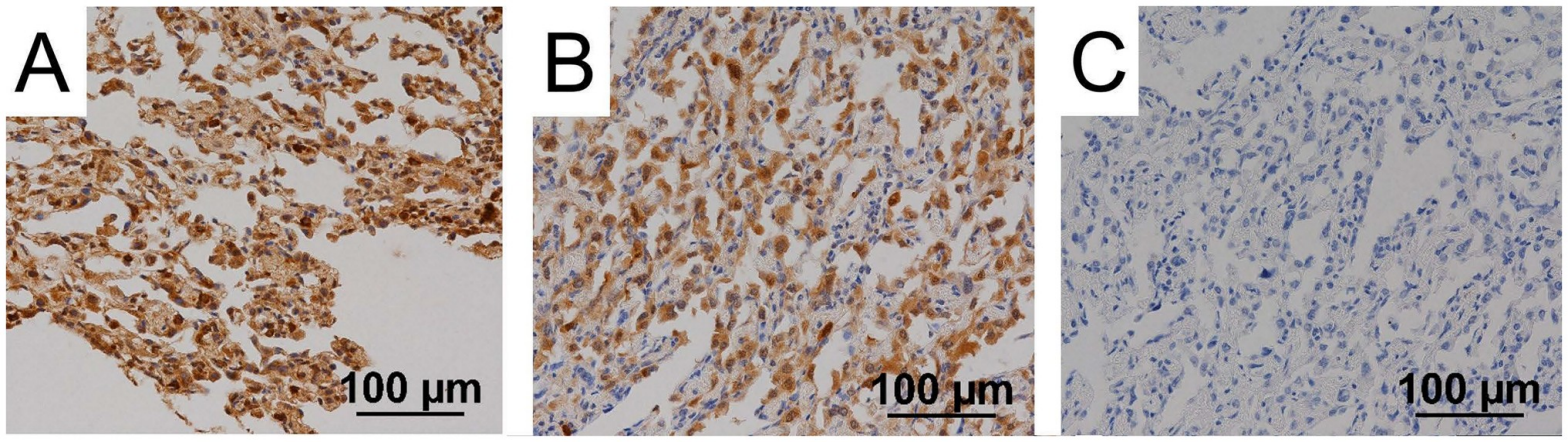
Immunohistochemical staining of lung lesions in patient 2. Multifocal micronodular pneumocyte hyperplasia (MMPH) lesions of patient 2 were assessed immunohistochemically. High-powered views (×200). Phospho-p70S6K (A) and phospho-4E-BP1 (B) were expressed in the cytoplasm of MMPH cells. Phospho-AKT was not expressed in MMPH cells (C).

We investigated whether the point mutation affected *TSC1* function via immunohistochemical examination of lung tissue from patients 1 and 2. Fig 6 shows the immunohistochemical images of samples from patient 2. *TSC1* is involved in the phosphatidylinositol 3-kinase/AKT/mammalian target of rapamycin pathway [27]. Hamartin, encoded by *TSC1*, and tuberin, encoded by *TSC2*, form a complex, which functions as a tumour suppressor by inhibiting mammalian target of rapamycin complex 1, which phosphorylates p70S6K and 4E-BP1. Phospho-p70S6K activates the ribosomal subunit protein S6 and leads to ribosomal recruitment and protein translation. 4E-BP1 inhibits the activity of eIF4E. Phospho-4E-BP1 releases eIF4E and initiates translation. In both patients 1 and 2, immunohistochemical examination revealed that phospho-p70S6K and phopho-4E-BP1 were expressed in the cytoplasm of MMPH cells (Fig 6A and 6B). These findings implied that the function of the hamartin/tuberin complex was compromised.

Phospho-AKT inhibits the formation of the hamartin and tuberin complex and promotes the phosphorylation of p70S6K and 4E-BP1. However, in both patients 1 and 2, immunohistochemical examination revealed that phospho-AKT was not expressed in MMPH cells (Fig 6C), suggesting that the observed phosphorylation of p70S6K and 4E-BP1 was caused not by phospho-AKT, but by the loss of function of the hamartin/tuberin complex. Therefore, the results of immunohistochemical examination are consistent with the possibility that *TSC1* function may be impaired by the novel gene mutation.

## Discussion

To our knowledge, this is the first report to describe familial aggregation of MMPH. Intron analysis revealed that all three patients shared a novel intronic point mutation causing splicing abnormalities and the functional loss of *TSC1*. Thus, MMPH could occur in families wherein individuals harbour the *TSC1* mutation. In addition, LOH was detected in all MMPH lesions examined and may therefore be involved in the development of MMPH.

We confirmed the absence of significant genetic mutation in exons of *TSC1/2* and detected a novel intronic point mutation that caused splicing anomalies. Notably, 9.5% and 16.2% of all mutations in *TSC1* and *TSC2*, respectively, are associated with splicing anomalies [28, 29]. Intronic mutations and mutations affecting splicing account for 40% of patients in whom mutations could not be detected via conventional genetic analysis [28]. The absence of pathogenic genomic alterations in the exons of 160 tumour-related genes upon next-generation sequencing further corroborates the finding that this *TSC1* intronic branch point mutation is the cause of the MMPH.

MMPH often co-occurs with TSC, a disorder with an autosomal-dominant inheritance pattern. However, familial aggregation of MMPH has not been reported. The pathomechanism of MMPH is unclear. Even patients with TSC who share the same mutations in *TSC1/2* often have different clinical presentations and outcomes [14, 15]. Therefore, some types of TSC lesions appear to require additional events in each organ, in addition to a mutation in one *TSC1/2* allele. As an example of additional events, LOH, chromosomal aberrations, methylation of promoter sequences, and mutations of other genes are assumed [30]. These additional events may not always occur with the inheritance of mutations in *TSC1*, which may be why MMPH does not generally accumulate in families. Inactivation of the remaining wild-type allele via LOH (the second hit) has been examined in detail for hamartoma development. We found that LOH occurred in all lung lesions examined, although there were differences in the degree of LOH among the lesions. Thus, LOH may contribute to MMPH pathogenesis. Similarly, LOH also occurs in most TSC-related renal angiomyolipomas (AML) with different degrees and may be involved in AML pathogenesis [17].

Immunohistochemical examination revealed that *TSC1* function might be impaired in MMPH cells. MMPH cells were positive for both phospho-p70S6K and phospho-4E-BP1. Moreover, normal type II alveolar epithelial cells were both positive and negative for phospho-p70S6K and phospho-4E-BP1 (S5 Fig). This may reflect the presence or absence of LOH and the level of *TSC1* function in each cell; positive cells may have two mutant alleles of *TSC1* owing to LOH. However, immunostaining is a rather poor quantitative modality, and the non-uniform staining in normal type II alveolar epithelial cells may result exclusively from technical issues. Overall, the results of our immunohistochemical examination are consistent with the possibility that *TSC1* function may be impaired by the novel gene mutation in MMPH cells.

In this family, patients 1 and 3 fulfilled the current diagnostic criteria of definitive TSC [8]; however, patient 2 had mild clinical features related to TSC. Therefore, we initially expected that patient 2 (the mother of patients 1 and 3) may exhibit mosaicism. However, the present analysis revealed that she also had the same mutation as patients 1 and 3 and that LOH occurred commonly in lung lesions in both patients 1 and 2. Although the reason underlying the different clinical features among the three patients and the involvement of LOH in this difference remains unclear, this finding is consistent with some previous reports revealing that a single genetic mutation in *TSC1/2* can lead to different phenotypes in multiple individuals [14, 15].

Radiologically and pathologically, AAH and well-differentiated adenocarcinoma are the most prominent differential diagnoses of multiple GGOs. In contrast, MMPH is rare and can usually be diagnosed when TSC-related clinical findings are accounted for. Histologically, MMPH is characterised by fibrous thickening and an increase in elastic fibres in the alveolar septa, which are features potentially distinct from AAH, suggesting that careful pathological examination is helpful for differentiating among AAH, adenocarcinoma, and MMPH [3]. At times, it may be impossible to collect lesions of a size sufficient for pathological evaluation. In such cases, clinical information, e.g. whether the patient has TSC, may support the pathological diagnosis.

MMPH is a mild disease [2, 31] and cannot be diagnosed unless CT is performed. Thus, familial aggregation may have been overlooked. Previously, MMPH was thought to have a lower frequency than lymphangioleiomyomatosis (LAM) in TSC [32]. However, a current report revealed that MMPH had a higher frequency [33] than LAM. The observed prevalence of MMPH may increase with advancements in imaging analyses, such as CT. Furthermore, MMPH has been reported to occur without TSC [2, 5]. However, in these cases, the entire body was not sufficiently assessed. In particular, TSC arising from a *TSC1* mutation is less symptomatic [34] and more likely to be overlooked, as for patient 1 in the present study, who was reported to constitute a case of MMPH not accompanied by TSC [25] (ref. in Japanese). Detailed whole-body assessment including brain MRI, collection of family history with an emphasis on possible TSC features, and genetic analysis including analysis of introns may be necessary in such less symptomatic patients.

TSC patients require lifelong follow-up for potentially life-threatening complications, such as AML, LAM, and subependymal giant cell astrocytoma [35, 36], although MMPH itself has a limited effect on respiratory symptoms and respiratory function [2, 31]. Thus, early detection of TSC is crucial for all patients. Patients with multiple micro-sized solid nodules and nodular GGOs observed on CT should be assessed systematically considering TSC.

## Conclusions

In this study, we report a case wherein MMPH occurred within a family with a novel *TSC1* mutation causing loss of function. This suggested that MMPH might occur in families wherein individuals harbour *TSC1* mutations. In addition, LOH was present in all MMPH lesions and may contribute to MMPH development.

## Supporting information

S1 Fig Full-length agarose gel electrophoresis of reverse transcription-polymerase chain reaction (RT-PCR) products corresponding to Fig 4A. Lane 2 is the negative control. Molecular grade water was used instead of template DNA. In lanes 3 and 4, the template DNA used in lane 1 was diluted to one-third of the original concentration. Lanes 5 and 6 do not contain template DNA.
(TIF)

S2 Fig Full-length agarose gel electrophoresis of polymerase chain reaction-restriction fragment length polymorphism (PCR-RFLP) products corresponding to Fig 5B. The portion surrounded by the white square frame was inverted horizontally and used in Fig 5B, which shows the PCR-RFLP for patient 2. Lane numbers coincide with those in Fig 5B. The samples from patient 1 were used for the lanes indicated by a white bar. However, the band images were not clear and were unsuitable for densitometric analysis. Therefore, PCR-RFLP for patient 1 was performed a second time. Mutation bands appeared in all lanes, and densitometric analysis was performed in the same way as in patient 2 (data not shown). Lanes indicated by a black bar contain no samples.
(TIF)

S3 Fig Full-length agarose gel electrophoresis of polymerase chain reaction-restriction fragment length polymorphism (PCR-RFLP) products corresponding to Fig 5C. Lane 8 contains half of the DNA used in lane 2 treated with half of the restriction enzyme compared to lanes 1 through 5. Lanes 6, 7, 9, and 10 did not contain a DNA sample.
(TIF)

S4 Fig Loss of heterozygosity (LOH) analysis of lung tissue from patient 1 (daughter). Polymerase chain reaction-based restriction fragment length polymorphism (PCR-RFLP) revealed that mutant alleles increased due to LOH in all lung lesions of patient 1 (daughter). (A) DNA was extracted from four lesions in paraffin-embedded lung tissue obtained via video-assisted thoracic surgery lung biopsy. Lesion 5 included scant multifocal micronodular pneumocyte hyperplasia (MMPH) cells, and lesions 6–8 included numerous MMPH cells. (B) PCR-RFLP analysis of lung lesions. Lane numbers match the lesion numbers in (A). In all four lanes, there were bands indicating 30- and 91-bp fragments cleaved by SspI. (C) Densitometry analysis of bands in lung lesion DNA. Letters indicating the area match those in (B). The area of each band determined via densitometric analysis is shown in S1 Table.
(TIF)

S5 Fig Immunohistochemical staining of normal lung specimens in patient 2. Non-multifocal micronodular pneumocyte hyperplasia (MMPH) parts of lung in patient 2 were assessed immunohistochemically. High-power views (x200). Phospho-p70S6K positive parts (A) and negative parts (B) were observed. Likewise for phospho-4E-BP1, there were positive parts (C) and negative parts (D).
(TIF)

S1 Table Densitometry analysis of lung lesions of Patient 1 (daughter). (DOCX)

S1 Dataset CEL nuclease-mediated heteroduplex incision with polyacrylamide gel electrophoresis and silver staining (CHIPS) analysis and long-range polymerase chain reaction (PCR) analysis for *TSC1* and *TSC2*. (XLS)

S2 Dataset Next-generation sequencing for 160 cancer-related genes including *TSC1* and *TSC2*. (XLSX)
