# Supplementary figures and images for: Familial multifocal micronodular pneumocyte hyperplasia with a novel splicing mutation in TSC1: Three cases in one family
Source: PLoS One. 2019 Feb 22;14(2):e0212370. doi: 10.1371/journal.pone.0212370 (PMC6386448; doi:10.1371/journal.pone.0212370)

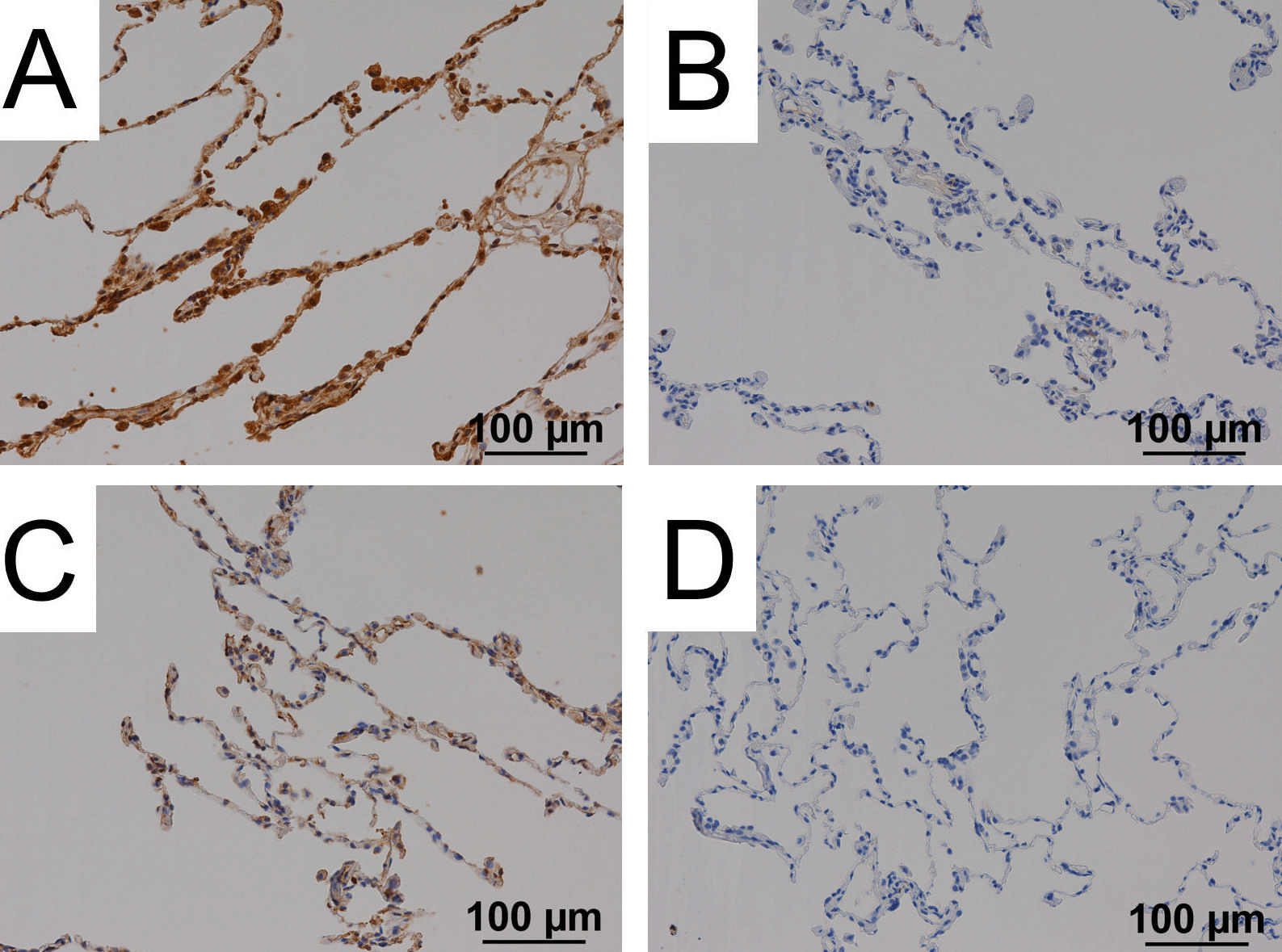

Supplement: S5 Fig — Non-multifocal micronodular pneumocyte hyperplasia (MMPH) parts of lung in patient 2 were assessed immunohistochemically. High-power views (x200). Phospho-p70S6K positive parts (A) and negative parts (B) were observed. Likewise for phospho-4E-BP1, there were positive parts (C) and negative parts (D). (TIF) [file pone.0212370.s005.tif]
