# Supplementary material for: Familial multifocal micronodular pneumocyte hyperplasia with a novel splicing mutation in TSC1: Three cases in one family
Source: PLoS One. 2019 Feb 22;14(2):e0212370. doi: 10.1371/journal.pone.0212370 (PMC6386448; doi:10.1371/journal.pone.0212370)
Supplement: S1 Table — (DOCX) [file pone.0212370.s006.docx]

**S1 Table. Densitometry analysis of lung lesions of Patient 1 (daughter).**

| Band | Lesion no. | Wild type  or mutation | Area | Area-Mut/Area-WT of each lesion |
| --- | --- | --- | --- | --- |
| o | 5 | Wild | 4402.569 |  |
| p | 5 | Mutation | 4795.175 | 1.089 |
| q | 6 | Wild | 5535.347 |  |
| r | 6 | Mutation | 4037.175 | 0.729 |
| s | 7 | Wild | 2302.548 |  |
| t | 7 | Mutation | 8805.246 | 3.824 |
| u | 8 | Wild | 4264.326 |  |
| v | 8 | Mutation | 8032.832 | 1.884 |

Letters indicating bands match those in S4 Fig B and C. Numbers indicating lesions match those in S4 Fig A and B.
